# Cardiac Biomarkers in Patients With Asymptomatic Severe Aortic Stenosis: Analysis From the EARLY TAVR Trial

**DOI:** 10.1161/CIRCULATIONAHA.125.074425

**Published:** 2025-03-31

**Authors:** Brian R. Lindman, Philippe Pibarot, Allan Schwartz, J. Bradley Oldemeyer, Yan Ru Su, Kashish Goel, David J. Cohen, William F. Fearon, Vasilis Babaliaros, David Daniels, Adnan Chhatriwalla, Hussam S. Suradi, Pinak Shah, Molly Szerlip, Michael J. Mack, Thom Dahle, William W. O’Neill, Charles J. Davidson, Raj Makkar, Tej Sheth, Jeremiah Depta, James T. DeVries, Jeffrey Southard, Andrei Pop, Paul Sorajja, Rebecca T. Hahn, Yanglu Zhao, Martin B. Leon, Philippe Généreux

**Affiliations:** Structural Heart and Valve Center (B.R.L.), Vanderbilt University Medical Center, Nashville, TN.; Division of Cardiovascular Medicine (B.R.L., Y.R.S., K.G.), Vanderbilt University Medical Center, Nashville, TN.; Cardiology Core Laboratory for Translational and Clinical Research (Y.R.S.), Vanderbilt University Medical Center, Nashville, TN.; Department of Cardiology, Québec Heart and Lung Institute, Laval University, Canada (P.P.).; Columbia University Medical Center/NewYork-Presbyterian Hospital, New York (A.S., D.J.C., R.T.H., M.B.L.).; University of Colorado Health, Loveland (J.B.O.).; Cardiovascular Research Foundation, New York, NY (D.J.C., M.B.L.).; St Francis Hospital and Heart Center, Roslyn, NY (D.J.C.).; Interventional Cardiology Section, Division of Cardiovascular Medicine, Department of Medicine, Stanford University School of Medicine, CA (W.F.F.).; VA Palo Alto Health Care System, CA (W.F.F.).; Emory School of Medicine, Atlanta, GA (V.B.).; Division of Cardiology, California Pacific Medical Center, San Francisco (D.D.).; St Luke’s Mid America Heart Institute, Kansas City, MO (A.C.).; Division of Cardiology, Rush University Medical Center, Chicago, IL (H.S.S.).; Division of Cardiovascular Medicine, Brigham and Women’s Hospital, Boston, MA (P. Shah).; Baylor Scott & White Heart Hospital, Plano, Richardson, TX (M.S., M.J.M.).; CentraCare Heart & Vascular Center, St Cloud, MN (T.D.).; Center for Structural Heart Disease, Henry Ford Health System, Detroit, MI (W.W.O.).; Northwestern University Feinberg School of Medicine, Chicago, IL (C.J.D.).; Smidt Heart Institute, Cedars-Sinai Medical Center, Los Angeles, CA (R.M.).; Population Health Research Institute, McMaster University and Hamilton Health Sciences, Hamilton, Ontario, Canada (T.S.).; Sands-Constellation Heart Institute, Rochester Regional Health, NY (J.D.).; Heart and Vascular Center, Section of Cardiovascular Medicine, Dartmouth-Hitchcock Medical Center, Lebanon, NH (J.T.D.).; Division of Cardiovascular Medicine, UC Davis Health System, University of California–Davis, Sacramento (J.S.).; Department of Cardiology, AMITA Alexian Brothers Medical Center, Elk Grove Village, IL (A.P.).; Minneapolis Heart Institute Foundation, Abbott Northwestern Hospital, MN (P. Sorajja).; Edwards Lifesciences, Irvine, CA (Y.Z.).; Gagnon Cardiovascular Institute, Morristown Medical Center, NJ (P.G.).

**Keywords:** aortic valve, aortic valve stenosis, biomarkers, natriuretic peptide, brain, transcatheter aortic valve replacement, troponin T, watchful waiting

## Abstract

**BACKGROUND:**

The EARLY TAVR trial (Evaluation of TAVR Compared to Surveillance for Patients With Asymptomatic Severe Aortic Stenosis) demonstrated that early transcatheter aortic valve replacement (TAVR) intervention was superior to clinical surveillance with delayed TAVR in patients with asymptomatic severe aortic stenosis. Cardiac biomarkers are associated with maladaptive remodeling, symptom onset, and worse outcomes after TAVR. Whether elevated biomarkers identify asymptomatic patients more likely to benefit from early intervention is unknown.

**METHODS:**

A core laboratory measured NT-proBNP (N-terminal pro–B-type natriuretic peptide) and high-sensitivity cardiac troponin T (hs-cTnT) levels. Associations between biomarker levels and risk of the trial primary end point (death, stroke, or unplanned cardiovascular hospitalization) and other secondary end points were examined with Kaplan-Meier curves and Cox proportional hazard models. Interaction tests were performed to assess whether the treatment effect of early TAVR, compared with clinical surveillance, differed according to biomarker levels.

**RESULTS:**

Among 901 patients randomized in EARLY TAVR, 798 (89%) had biospecimens measured (median NT-proBNP level, 287 [145, 601]; median hs-cTnT level, 14.6 [10.5, 21.0]). Higher levels of NT-proBNP and hs-cTnT were broadly associated with higher event rates for multiple end points. In general, there was no significant interaction between baseline biomarkers and treatment group with respect to any composite or individual end point examined, although trends broadly demonstrated a greater relative benefit of early TAVR at lower biomarker levels. There was a significant interaction between hs-cTnT level and treatment group with respect to death or heart failure hospitalization (*P*_interaction_=0.04) and heart failure hospitalization alone (*P*_interaction_=0.03) such that the relative benefit of early TAVR was greater for patients with normal, rather than elevated, levels of hs-cTnT at baseline. For some end points, higher baseline NT-proBNP level was associated with numerically greater absolute risk reduction with early TAVR than were lower NT-proBNP levels.

**CONCLUSIONS:**

In patients with asymptomatic severe high-gradient aortic stenosis, higher NT-proBNP and hs-cTnT levels were broadly associated with higher event rates, as expected. However, the relative benefit of an early TAVR strategy was consistent regardless of baseline biomarker levels and, contrary to our hypothesis, tended to be more pronounced in patients with the lowest biomarker levels. These findings suggest limited value for single measurements of these biomarkers to guide the timing of TAVR in asymptomatic patients.

**REGISTRATION:**

URL: https://www.clinicaltrials.gov; Unique identifier: NCT03042104.

**Clinical Perspective**

**What Is New?**

- For the first time in the context of a randomized strategy trial testing the effect of prompt transcatheter aortic valve replacement (TAVR) compared with clinical surveillance in patients with asymptomatic severe high-gradient aortic stenosis, this subgroup analysis of the EARLY TAVR trial (Evaluation of TAVR Compared to Surveillance for Patients With Asymptomatic Severe Aortic Stenosis) examined whether increased NT-proBNP (N-terminal pro–B-type natriuretic peptide) or hs-cTnT (high-sensitivity cardiac troponin T) levels identify a subgroup of patients who benefit more from an early intervention strategy.
- These data show a generally consistent treatment effect of early TAVR compared with clinical surveillance across biomarker subgroups.

**What Are the Clinical Implications?**

- The findings suggest limited value for single measurements of these biomarkers to guide the timing of TAVR in asymptomatic patients.
- Even when biomarker levels were low or normal, there was a relative benefit to early TAVR that, counter to our hypothesis, tended to be greater than among patients with higher biomarker levels.
- When biomarker levels are elevated, there is even more urgency to intervene promptly given higher event rates among these patients and greater absolute risk reduction in patients with higher NT-proBNP levels for end points, including heart failure hospitalization.

**Editorial, see p 1565**

For patients with symptomatic severe aortic stenosis (AS), prompt aortic valve replacement (AVR) is strongly recommended, and there is no role for other testing or factors to tailor the timing of the intervention.^1^ However, as outlined in guidelines, a more nuanced approach has been recommended in asymptomatic patients with severe AS. For example, in stage C1 cases with high gradients, the guidelines indicate that AVR is reasonable (2a recommendation) in patients with a natriuretic peptide level >3 times normal.^1^

There are accumulating data linking maladaptive left ventricular (LV) remodeling or dysfunction before AVR with worse postprocedural outcomes and a higher residual risk related predominantly to heart failure.^2–5^ Previous studies have revealed that elevated biomarkers of cardiac stress (eg, natriuretic peptides) and damage (eg, high-sensitivity troponin) are associated with maladaptive LV remodeling or dysfunction, earlier symptom onset, and worse outcomes after AVR.^6–10^ These studies provide the rationale for the guideline recommendation that AVR is reasonable in patients with asymptomatic severe AS and elevated BNP (brain natriuretic peptide) to unload the heart to potentially prevent irreversible cardiac damage and its consequences. However, this recommendation is based on nonrandomized, observational data.^1^ In clinical practice, these biomarkers are often measured in patients with AS who lack symptoms or who have symptoms of unclear pathogenesis as an adjunctive manner of monitoring disease progression and informing timing of AVR.

The EARLY TAVR trial (Evaluation of TAVR Compared to Surveillance for Patients With Asymptomatic Severe Aortic Stenosis) recently tested the hypothesis that in patients with asymptomatic severe AS (stage C1), prompt intervention with transcatheter AVR (TAVR) leads to improved outcomes compared with clinical surveillance (CS) with delayed TAVR when symptoms or another guideline indication for AVR develop.^11^ The trial demonstrated that early TAVR significantly reduced the rates of the primary end point composite and several secondary outcomes. The trial protocol also included prospective collection of biospecimens enabling a prespecified analysis to determine whether cardiac biomarker levels (ie, NT-proBNP [N-terminal pro-B–type natriuretic peptide], hs-cTnT [high-sensitivity cardiac troponin T]) may be useful to identify a subgroup of patients who may benefit more from early AVR, a hypothesis that has never been tested in a randomized strategy trial on AVR timing. We hypothesized that patients with elevated biomarker levels would have higher event rates and would experience greater benefit from early TAVR compared with patients with lower biomarker levels.

## METHODS

### Data Availability Statement

Because of the sensitive nature of the data collected for this study, requests to access the data set from qualified researchers trained in clinical trials and human subject confidentiality protocols should be sent to the corresponding author.

### Trial Design and Patient Population

The trial design and rationale have been described previously, and have been approved by the Food and Drug Administration as well as by the institutional review board at each site.^11,12^ EARLY TAVR is a prospective, multicenter, open-label, randomized controlled trial in which upfront TAVR with transfemoral placement of a SAPIEN 3 or SAPIEN 3 Ultra valve (Edwards Lifesciences) was compared with CS and deferred TAVR among patients with asymptomatic severe AS and indications for CS under current guidelines. Eligible patients who provided written informed consent were enrolled and randomized in a 1:1 ratio to early TAVR or CS. Randomized patients were considered part of the intention-to-treat (ITT) population.

### Biomarker Measurements

Plasma specimens were collected at baseline by the site from patients in the EARLY TAVR trial and shipped on dry ice to the core laboratory. NT-proBNP (proBNP II immunoassay) and hs-cTnT (Troponin T Gen 5 STAT) levels were measured by a core laboratory using Food and Drug Administration–approved Roche Elecsys assays on the Cobas e411 immunoassay analyzer (Roche Diagnostics). The NT-proBNP assay has a measuring range of 5 to 35 000 pg/mL. The hs-cTnT assay has a measuring range of 3 to 10 000 pg/mL. The precision for both assays is <2%. The time between biospecimen collection and performance of TAVR in the early TAVR arm was brief (median, 24 days [interquartile range, 8–43 days]).

### Analysis End Points

The primary end point of the EARLY TAVR trial was a nonhierarchical composite of all-cause death, stroke, or unplanned cardiovascular hospitalization (including aortic valve intervention or reintervention within 6 months of randomization).^11^ Secondary composite end points were as follows: (1) all-cause death, all stroke, or heart failure hospitalization (HFH); (2) all-cause death or unplanned cardiovascular hospitalization (including aortic valve intervention or reintervention within 6 months); and (3) all-cause death, all stroke, unplanned cardiovascular hospitalization, or intervention or reintervention with advanced signs or symptoms. As defined previously, for this last composite end point, aortic valve intervention within 6 months of randomization was not considered an unplanned cardiovascular hospitalization unless it was accompanied by advanced signs or symptoms. Individual components of these composites were also examined, including HFH, cardiovascular death, and new-onset atrial fibrillation. HFH was defined as follows: hospitalization for clinical symptoms of congestive heart failure with objective signs and administration of intravenous diuresis or inotropic therapy, institution of mechanical support (intra-aortic balloon pump or ventilation for pulmonary edema), or hemodialysis for volume overload. All end points were adjudicated by the clinical events committee. In the CS patient arm, timing of conversion to AVR (delayed AVR) and type of presentation at the time of conversion (ie, advanced or acute signs and symptoms, progressive signs and symptoms, or no symptoms, as defined previously)^11^ were examined in each biomarker subgroup. All end points were adjudicated by the clinical events committee according to prespecified definitions. Members of the clinical events committee were not blinded to the treatment arm to which patients were randomized.

### Statistical Analysis

For analytic purposes, patients were stratified according to baseline biomarker levels as follows: (1) first, second, and third tertiles for each absolute biomarker concentration; (2) normal versus elevated biomarker levels; and (3) number of elevated biomarker levels (0, 1, or 2). As recommended in the assay package inserts, elevated NT-proBNP level was defined as ≥125 pg/mL for patients <75 years of age and ≥450 pg/mL for patients ≥75 years of age. Elevated hs-cTnT level was defined as ≥14 pg/mL for women and ≥22 pg/mL for men. The biomarkers were also log-transformed when analyzed as continuous variables.

Continuous variables are presented as mean±SD or median (25th percentile, 75th percentile) and categorical and binary variables are presented as count (%). Continuous and ordinal variables were compared using the Jonckheere-Terpstra test among ordinal biomarker groups and the Wilcoxon rank sum test between binary biomarker groups; binary outcomes were compared using the Cochran-Armitage test among ordinal biomarker groups and the Fisher Exact test between binary biomarker groups. Time-to-event outcomes were reported as Kaplan-Meier curves as well as incidence rate per 100 person-years. Associations of biomarker levels with clinical outcomes were examined with Kaplan-Meier curves as well as Cox proportional hazard regression models (unadjusted and adjusted for baseline covariates: age, sex, aortic valve peak velocity, cerebrovascular accident, diabetes, coronary artery disease, estimated glomerular filtration rate, atrial fibrillation, and moderate or greater mitral regurgitation); proportional hazard assumption was checked with a Schoenfeld residuals plot. The treatment effect of early TAVR versus CS within each biomarker subgroup was examined and a potential differential effect was evaluated in Cox proportional hazard models with an interaction term (treatment group x biomarker subgroup); in addition, numbers needed to treat (NNTs) at 2 years after randomization were reported by biomarker subgroups. A 2-sided *P*<0.05 was considered statistically significant (without correction for multiple comparisons). All statistical analyses were performed with the use of SAS software, version 9.4 (SAS Institute).

## RESULTS

### Patient Characteristics

Biomarker data were available for 798 randomized patients in the ITT population (TAVR arm, n=414; CS arm, n=384; Figure S1). Baseline characteristics by NT-proBNP and hs-cTnT tertiles are shown in Table 1. Higher NT-proBNP and hs-cTnT levels were associated with older age, higher Society of Thoracic Surgeons risk score, inability to perform a treadmill stress test, shorter 6-minute walk distance, a greater burden of atrial fibrillation, and worse renal function. Higher NT-proBNP levels were associated with lower body mass index and less diabetes but more previous stroke; higher hs-cTnT levels were associated with a higher burden of coronary artery disease, including previous MI, and diabetes. Kansas City Cardiomyopathy Questionnaire overall score was similar across tertiles of both biomarkers.

**Table 1.**
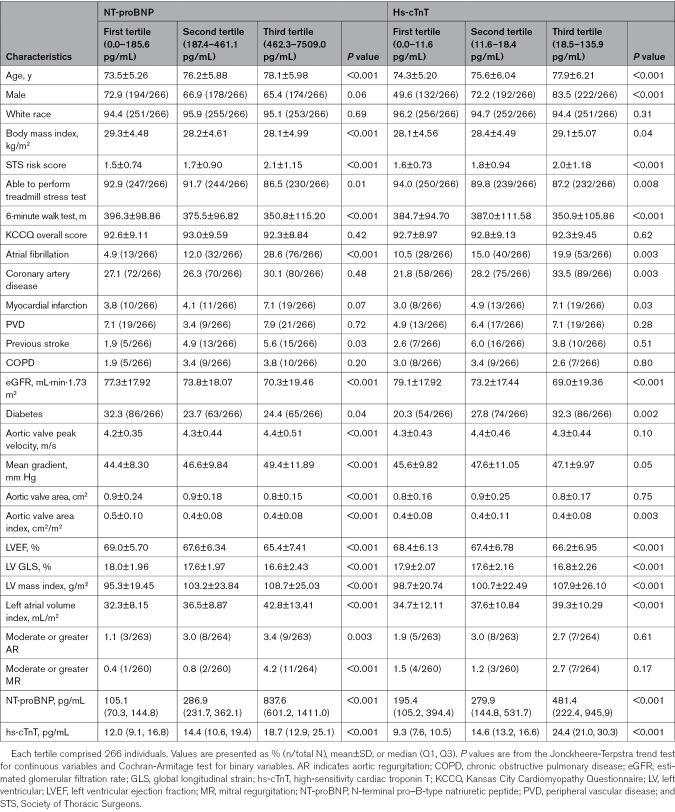
Baseline Characteristics by Screening NT-proBNP and hs-cTnT Level

With respect to baseline echocardiographic measures, higher NT-proBNP and hs-cTnT levels were associated with more severe AS (more consistently across all hemodynamic indices for NT-proBNP), worse LV systolic and diastolic function, and higher LV mass index. Higher NT-proBNP levels were also associated with more frequent moderate or moderate to severe aortic regurgitation and mitral regurgitation .

When patients were stratified according to elevated versus normal levels of NT-proBNP and hs-cTnT, or counts of elevated biomarkers (0, 1, or 2 elevated), there were similar trends in baseline characteristics and demographics as in the tertile subgroup analysis (Tables S1 and S2).

### Biomarkers and Event Rates

**Figure 1.**
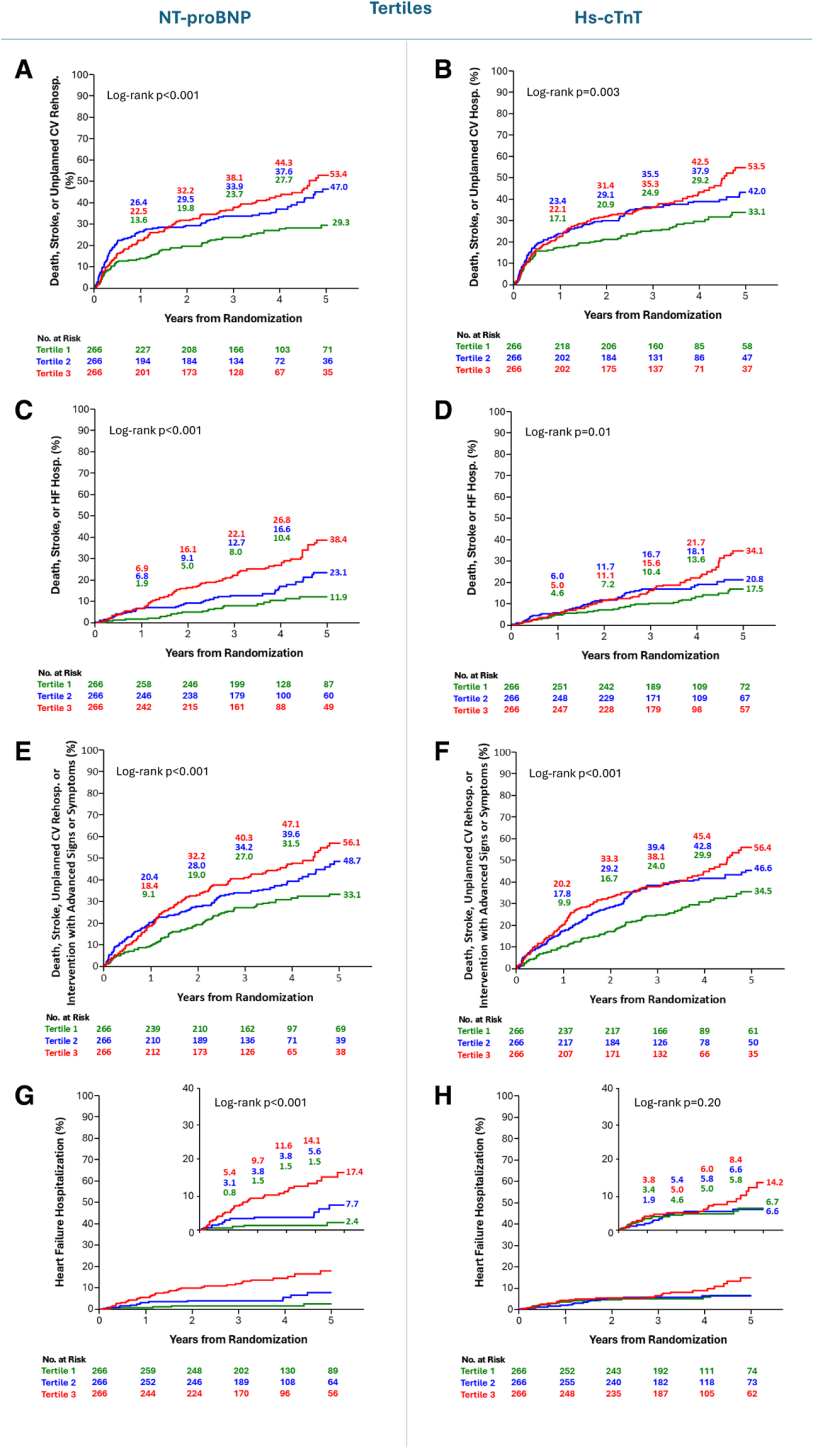
**Kaplan-Meier curves for the key clinical outcomes according to NT-proBNP and hs-cTnT tertiles.** Kaplan-Meier curves for the composites of all-cause death, all stroke, or unplanned cardiovascular hospitalization: **A**, NT-proBNP (N-terminal pro–B-type natriuretic peptide); **B**, hs-cTnT (high-sensitivity cardiac troponin T); all-cause death, all stroke, or heart failure (HF) hospitalization; **C**, NT-proBNP; **D**, hs-cTnT; all-cause death, all stroke, unplanned cardiovascular hospitalization, or intervention or reintervention with advanced signs or symptoms; **E**, NT-proBNP; **F**, hs-cTnT; and HF hospitalization; **G**, NT-proBNP; and **H**, hs-cTnT. The insets in **E** and **F** show the same data on an enlarged *y* axis. *P* value shown is based on an unadjusted log-rank test with data up to 5 years. Tertile 1 is shown in green, tertile 2 is shown in blue, and tertile 3 is shown in red.

Higher concentrations of NT-proBNP and hs-cTnT, examined as tertiles, were associated with higher event rates for the primary and most secondary end points of the trial among patients in the ITT population (Figure 1; Figure S2). Kaplan-Meier curves and event rates for these end points based on elevated versus normal NT-proBNP levels, elevated versus normal hs-cTnT levels, and number of biomarkers elevated (0, 1, or 2) demonstrated similar associations, with higher biomarker levels generally associated with higher event rates (Figures S3 through S5).

In unadjusted and most adjusted analyses, higher NT-proBNP level (as a continuous variable) was associated with significantly higher event rates among patients in the ITT population (Table 2). In contrast, higher hs-cTnT level (as a continuous variable) was not associated with higher event rates after adjustment (Table 2). Unadjusted and adjusted associations stratified by treatment group are shown in Table S3. In contrast, when examined as elevated versus normal levels, elevated NT-proBNP level was less consistently associated with worse outcomes after adjustment, whereas elevated hs-cTnT level was associated with higher event rates for many end points after adjustment in the ITT population (Table S4). When examined as the number of biomarkers elevated, 2 elevated biomarkers, compared with 0, was associated with higher event rates for most end points in the ITT population after adjustment; similar associations were seen for several end points for patients with 1 elevated biomarker (Table S5).

**Table 2.**
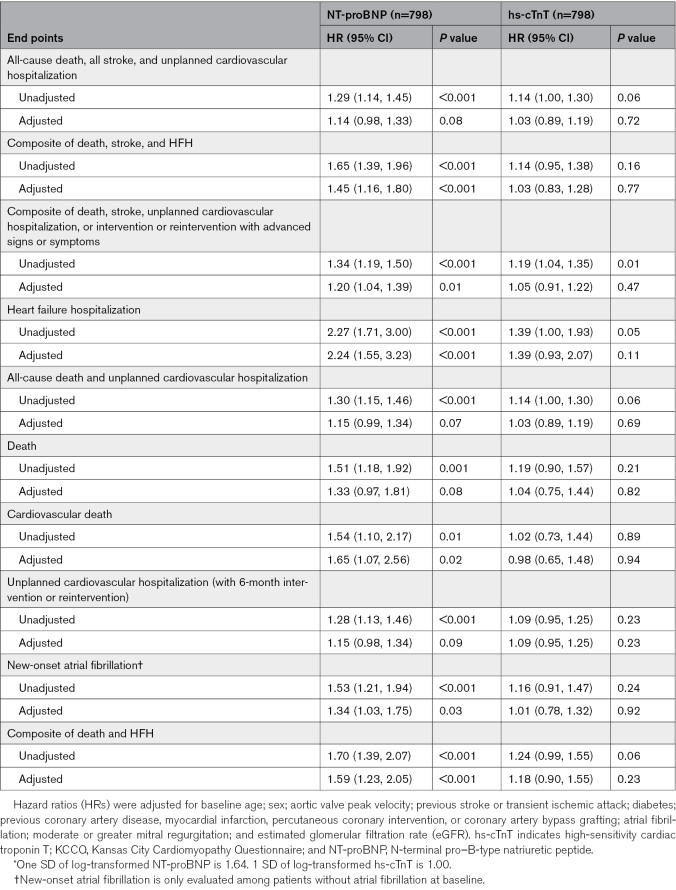
Hazard Ratio per 1 SD* Increase in Log-Transformed NT-proBNP or hs-cTnT

### Early TAVR Versus CS by Circulating Biomarker Concentration

**Figure 2.**
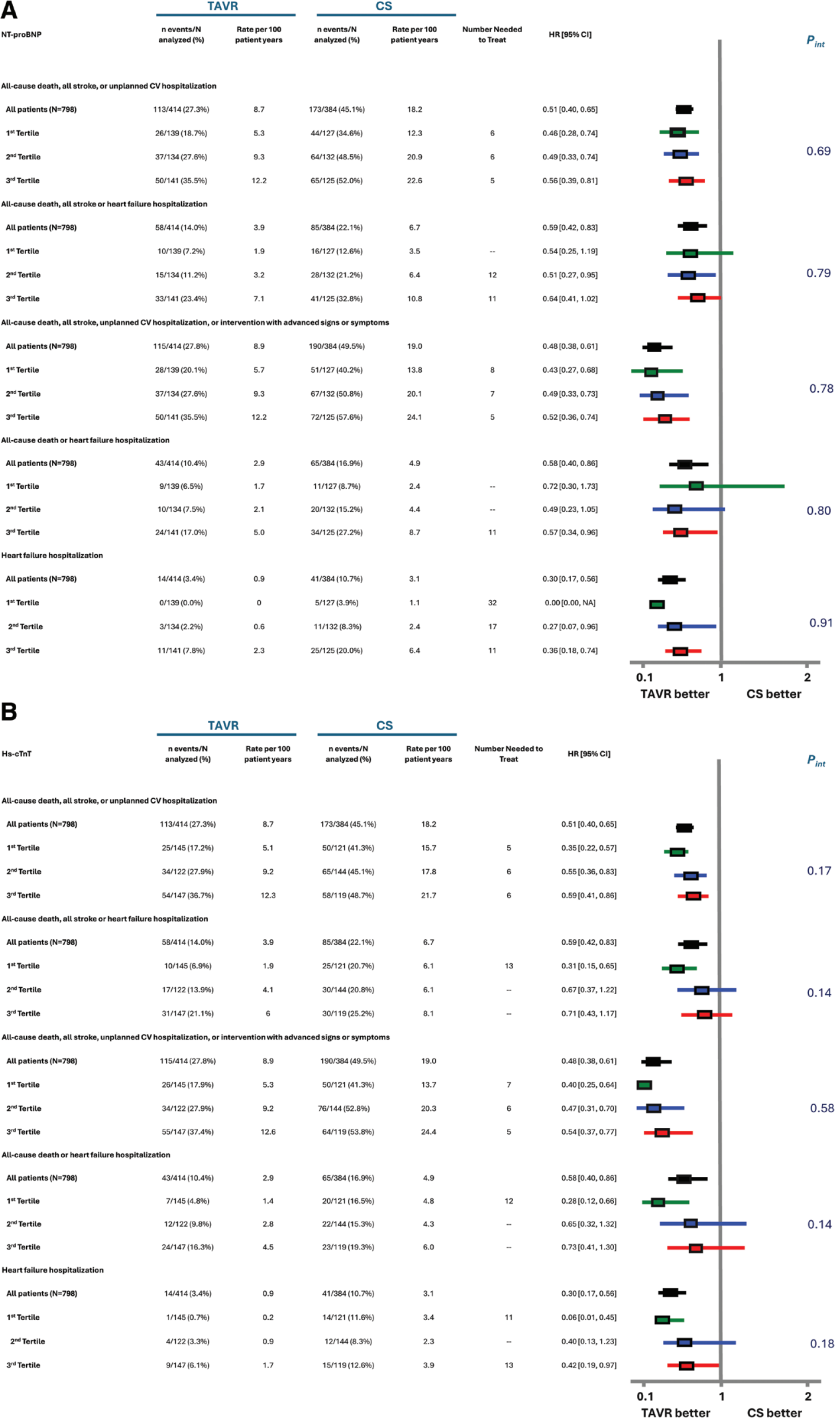
**Forest plot showing key clinical outcome event rates and treatment effect according to NT-proBNP and hs-cTnT tertiles.** Forest plot showing key clinical outcome event rates and treatment effect according to NT-proBNP (N-terminal pro–B-type natriuretic peptide; **A**) or hs-cTnT (high-sensitivity cardiac troponin T; **B**) tertiles. Clinical event incidence (n/N) and Kaplan-Meier rates, rates per 100 patient-years, number needed to treat, risk, and interaction *P* values are shown for all-cause death, all stroke, or unplanned cardiovascular hospitalization; all-cause death, all stroke, or heart failure hospitalization; all-cause death, all stroke, unplanned cardiovascular hospitalization, or intervention or reintervention with advanced signs or symptoms; all-cause death or heart failure hospitalization; and heart failure hospitalization according to all patients included in the biomarker analysis (n=798) and tertiles 1, 2, or 3 for NT-proBNP (**A**) or hs-cTnT (**B**) levels. Number needed to treat values are presented when transcatheter aortic valve replacement (TAVR) has a statistically significantly lower hazard ratio (HR) compared with clinical surveillance (CS). Clinical event HRs and 95% confidence limits are based on Cox proportional hazard models with data up to 5 years. Interaction *P* values (*P*_interaction_) reflect interaction between tertile and treatment arm. Tertile 1 is shown in green, tertile 2 is shown in blue, and tertile 3 is shown in red.

For the primary end point of the trial and for several key secondary end points, there were no significant interactions between NT-proBNP nor hs-cTnT biomarker tertiles and treatment group assignment (Figure 2; Figure S6). The relative benefit of early TAVR, compared with CS, was similar regardless of the biomarker tertile. Whereas not statistically significant, there was a trend for greater benefit of early TAVR compared with CS for most end points examined (ie, the hazard ratio [HR] was numerically lower) among patients in the lowest biomarker tertile. Event rates were consistently lowest for key end points examined among patients in the lowest biomarker tertile undergoing early TAVR (Figure 2). Similar relationships were generally observed when subgroup analyses based on age (Table S6) and sex (Table S7) were performed, with no significant interactions (or trends) seen between biomarker tertile and treatment group with respect to outcomes.

Across NT-proBNP and hs-cTnT biomarker tertiles, differences in absolute event rates between the early TAVR and CS groups and the NNT were fairly similar for most end points, albeit with a larger difference across tertiles of NT-proBNP for the end point of HFH (Figure 2). For HFH, the NNT was 32 for the lowest NT-proBNP tertile and 11 for the highest NT-proBNP tertile (Figure 2).

**Figure 3.**
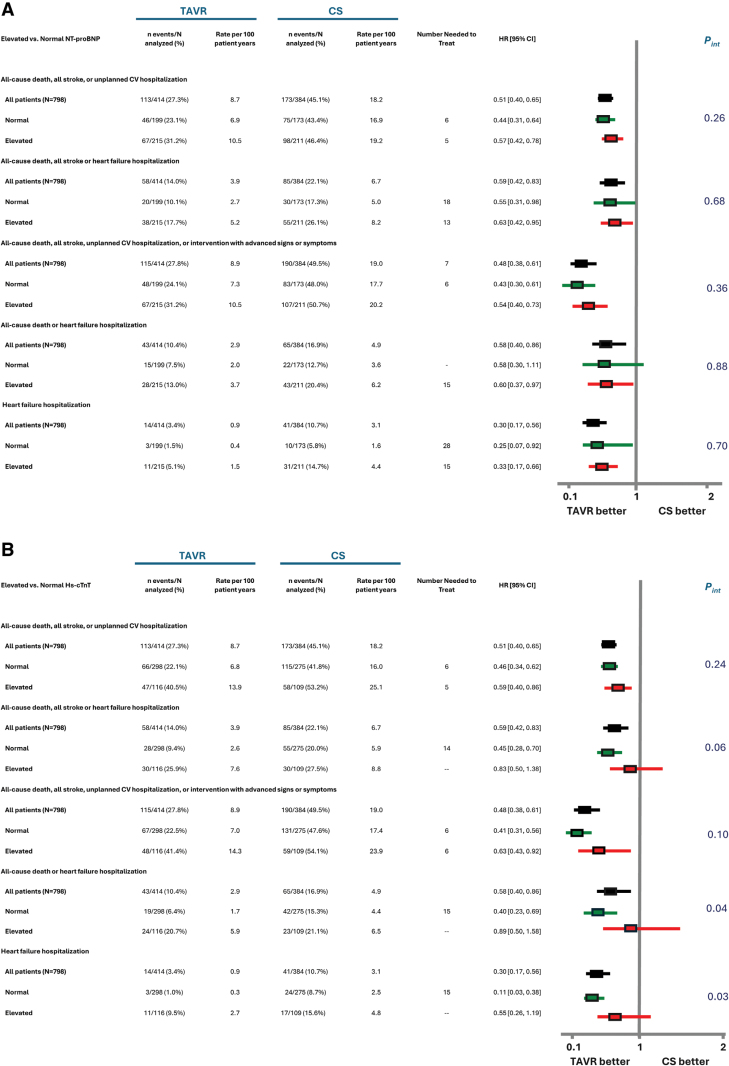
**Forest plot showing key clinical outcome event rates and treatment effect according to elevated vs normal NT-proBNP and hs-cTnT levels.** Forest plot showing key clinical outcome event rates and treatment effect according to elevated vs normal NT-proBNP (N-terminal pro–B-type natriuretic peptide; **A**) or hs-cTnT (high-sensitivity cardiac troponin T; **B**) levels. Clinical event incidence (n/N) and Kaplan-Meier rates, rates per 100 patient-years, number needed to treat, risk, and interaction *P* values are shown for all-cause death, all stroke, or unplanned cardiovascular hospitalization; all-cause death, all stroke, or heart failure hospitalization; all-cause death, all stroke, unplanned cardiovascular hospitalization, or intervention or reintervention with advanced signs or symptoms; all-cause death or heart failure hospitalization; and heart failure hospitalization according to all patients included in the biomarker analysis (n=798) and elevated vs normal levels of NT-proBNP (**A**) or hs-cTnT (**B**) levels. Number needed to treat values are presented when transcatheter aortic valve replacement (TAVR) has a statistically significantly lower hazard ratio (HR) compared with clinical surveillance (CS). Clinical event HRs and 95% confidence limits are based on Cox proportional hazard models with data up to 5 years. Interaction *P* values (*P*_interaction_) reflect interactions between the biomarker level and treatment arm. Normal is shown in green, and elevated is shown in red. Elevated NT-proBNP level is defined as ≥125 pg/mL for patients <75 years of age and ≥450 for patients ≥75 years of age. Elevated hs-cTnT level is defined as ≥14 pg/mL for women and ≥22 pg/mL for men.

**Figure 4.**
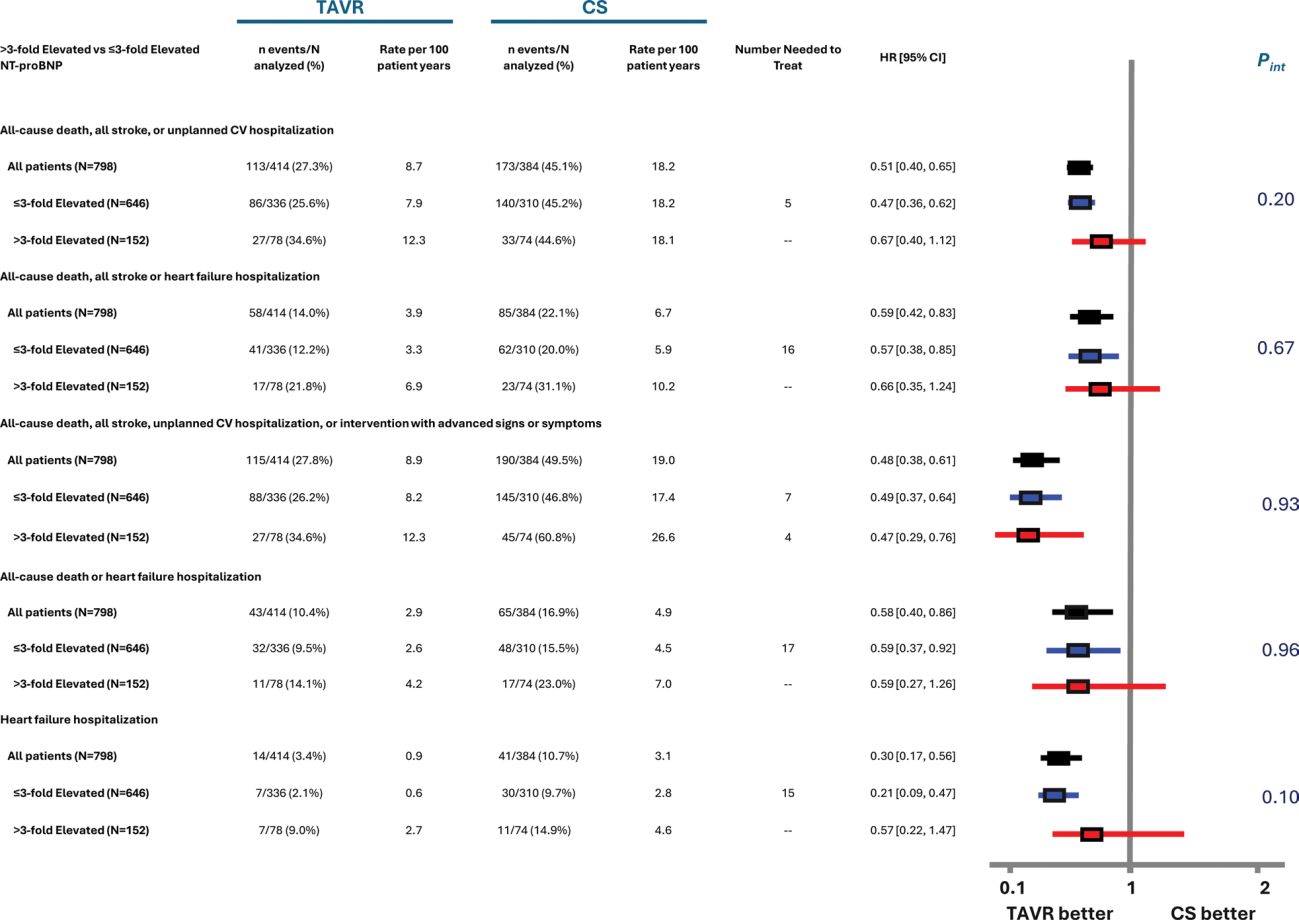
**Forest plot showing key clinical outcome event rates and treatment effect according to NT-proBNP level ≤3-fold elevated vs >3-fold elevated.** Clinical event incidence (n/N) and Kaplan-Meier rates, rates per 100 patient-years, number needed to treat, risk, and interaction *P* values are shown for all-cause death; all stroke or unplanned cardiovascular hospitalization; all-cause death, all stroke, or heart failure hospitalization; all-cause death, all stroke, unplanned cardiovascular hospitalization, or intervention or reintervention with advanced signs or symptoms; and heart failure hospitalization according to all patients included in the biomarker analysis (n=798), ≤3-fold elevated NT-proBNP (N-terminal pro–B-type natriuretic peptide) level (n=646), and >3-fold elevated NT-proBNP level (n=152). Number needed to treat values are presented when transcatheter aortic valve replacement (TAVR) has statistically significantly lower hazard ratio (HR) compared with clinical surveillance (CS). Clinical event HRs and 95% confidence limits are based on Cox proportional hazard models with data up to 5 years. Interaction *P* values (*P*_interaction_) reflect interactions between the tertile and treatment arm. The ≤3-fold elevated NT-proBNP level is shown in blue, and the >3-fold elevated NT-proBNP level is shown in red. Elevated NT-proBNP level is defined as ≥125 pg/mL for patients <75 years of age and ≥450 for patients ≥75 years of age.

Similar patterns were observed when NT-proBNP and hs-cTnT levels were evaluated as elevated versus normal (Figure 3). However, the interaction between hs-cTnT level (elevated versus normal) and treatment group was borderline significant for the composite end point of death, stroke, or HFH (*P*_interaction_=0.06) and significant for the composite of death or HFH (*P*_interaction_=0.04) and HFH alone (*P*_interaction_=0.03; Figure 3). Whereas the point estimate favored early TAVR for both end points regardless of whether hs-cTnT was elevated or not, the relative benefit of early TAVR was greater among patients with normal hs-cTnT levels. No significant interactions between NT-proBNP level (elevated to >3 times normal versus ≤3 times normal) and treatment group with respect to several clinical end points and the numerically lower HRs were generally observed among patients with NT-proBNP ≤3 times normal (Figure 4).

There were no significant interactions (although some were of borderline significance) on the basis of the number of elevated biomarkers and treatment group with respect to outcomes (Figure S7). Again, the benefit of early TAVR was numerically most pronounced in patients with normal NT-proBNP and hs-cTnT levels.

### Biomarkers and Delayed AVR Conversion in the CS Arm

**Figure 5.**
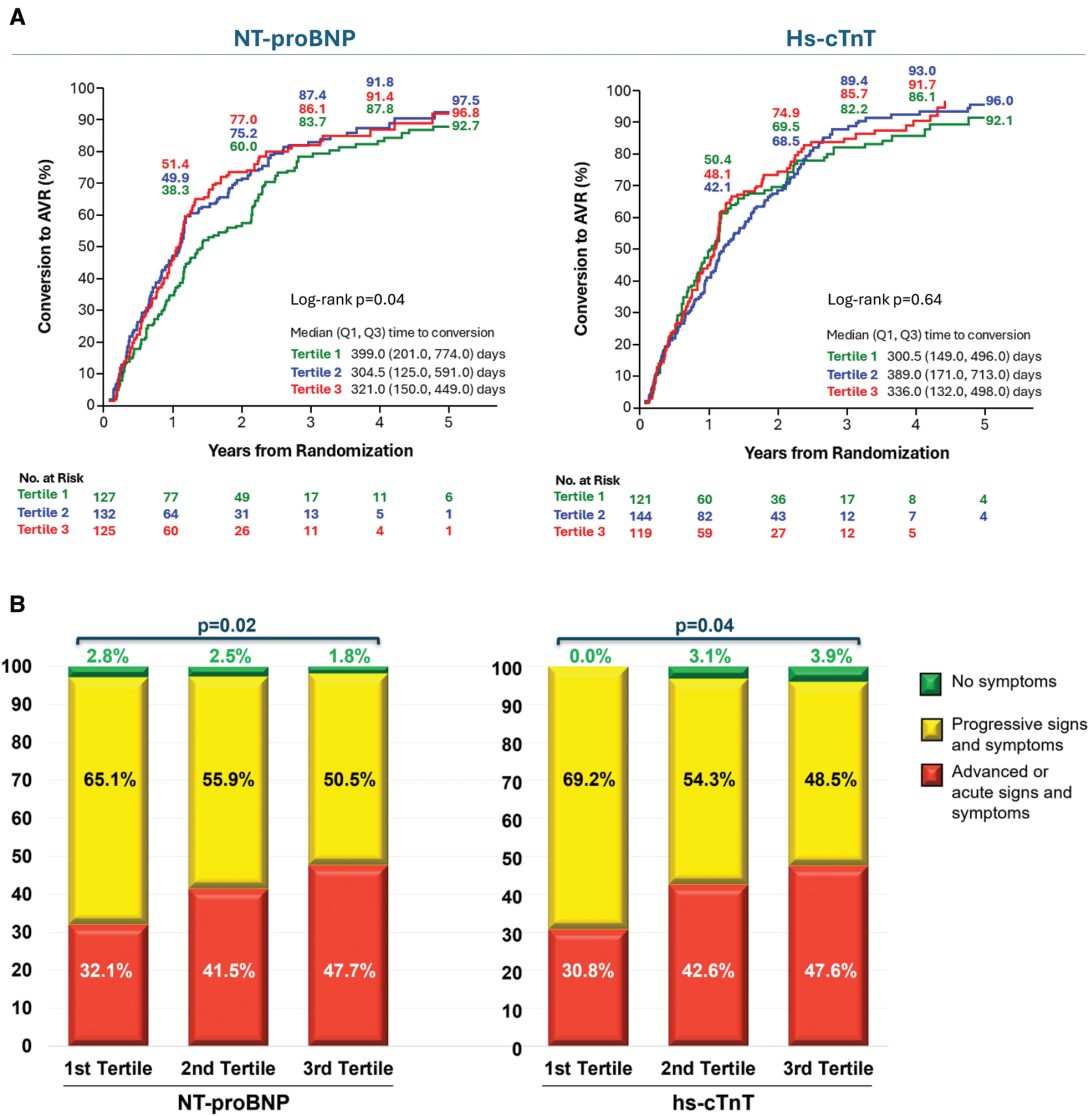
**Timing and clinical presentation class of AVR conversion in the CS arm according to biomarker tertiles. A**, Kaplan-Meier curves for the timing of conversion to aortic valve replacement (AVR) in patients randomized to the clinical surveillance (CS) arm according to tertile of NT-proBNP (N-terminal pro–B-type natriuretic peptide; **left**) and hs-cTnT (high-sensitivity cardiac troponin T; **right**). The median time to conversion and interquartile range (in days) by tertile is shown on each curve. Tertile 1 is shown in green, tertile 2 is shown in blue, and tertile 3 is shown in red. **B**, Stacked bar graphs showing the clinical presentation class at time of AVR conversion by NT-proBNP and hs-cTnT tertiles in patients randomized to the CS arm. *P* values shown are based on Jonckheere-Terpstra trend tests.

Among patients in the CS arm, there was a nominal difference in the timing of crossover to delayed AVR based on NT-proBNP tertiles driven by a modestly longer time to delayed AVR among patients in the lowest NT-proBNP tertile (Figure 5A). There was no difference in the timing of crossover to delayed AVR based on hs-cTnT tertiles (Figure 5A). Increasing NT-proBNP and hs-cTnT tertiles were associated with a higher proportion of an AVR conversion presentation with acute or advanced signs and symptoms, but such a presentation still occurred one-third of the time among patients in the lowest biomarker tertiles (Figure 5B). In patients stratified according to elevated versus normal biomarker levels, the median conversion time to AVR was modestly shorter from time of randomization in patients with elevated baseline NT-proBNP levels (315 days for elevated NT-proBNP versus 397 days for normal NT-proBNP) and elevated hs-cTnT (306 days for elevated hs-cTnT versus 347 days for normal hs-cTnT; Figure S8A). A higher number of counts of elevated biomarkers (0, 1, or 2) was also associated with a somewhat shorter time to AVR conversion (Figure S9A).

## DISCUSSION

Leveraging prospective biospecimen collection on 89% of participants in the largest randomized strategy trial on optimal timing of AVR in patients with asymptomatic severe AS, this report substantively advances our understanding of the potential role of NT-proBNP and hs-cTnT levels in clinical decision making. As expected, higher levels of NT-proBNP and hs-cTnT were associated with higher event rates for the primary and multiple secondary end points of the trial. The beneficial treatment effect of early TAVR, compared with CS, was consistent for the primary and multiple secondary end points regardless of baseline biomarker concentrations. This was true whether biomarkers were analyzed as tertiles, elevated versus normal, or based on the number of biomarkers elevated. There was a numeric trend toward a lower HR (indicative of a larger relative benefit of early TAVR) among patients with the lowest, rather than highest, biomarker levels, which ran contrary to our hypothesis. In 2 instances, there was a significant interaction between hs-cTnT level (elevated versus normal) and treatment group with respect to the composite of death or HFH and for HFH alone; whereas both hs-cTnT subgroups had a lower hazard of HFH with early TAVR, the HR was significantly lower in patients with a normal hs-cTnT level, again contrary to our hypothesis. Event rates were lowest across all end points when biomarker levels were low and patients were treated with an early TAVR strategy. NT-proBNP and hs-cTnT levels did not prove particularly useful in predicting timing of AVR conversion; in some cases, a higher biomarker level was associated with a higher proportion of an acute or severe presentation at AVR conversion, but one-third of CS patients in the lowest biomarker tertiles still had an acute or severe presentation at conversion. Biomarkers of cardiac stress or damage (either NT-proBNP or hs-cTnT, or other biomarkers) may identify a subgroup of patients with earlier stage AS who may benefit from early AVR, but among patients with asymptomatic high-gradient severe AS, NT-proBNP and hs-cTnT levels do not appear to be particularly useful for guiding clinical decision making on the timing of TAVR.

Previous work has demonstrated that, in patients with AS, the circulating natriuretic peptide concentration is associated with myocardial inflammation,^13^ hypertrophic remodeling,^9^ and impaired LV systolic function.^8^ From a clinical standpoint, higher natriuretic peptide levels have been associated with earlier symptom onset among asymptomatic patients,^10^ AS-related death or HFH in patients with asymptomatic severe AS not undergoing AVR,^14^ and survival in asymptomatic and symptomatic patients.^6,8,9^ Similarly, the circulating concentration of high-sensitivity troponin is associated with hypertrophic remodeling, diffuse and replacement fibrosis, and impaired LV systolic function.^7–9,13,15^ Moreover, higher circulating troponin levels are associated with death in patients with severe AS.^8,9^

Our results confirm this previous work by demonstrating that higher NT-proBNP and hs-cTnT levels at baseline were associated with more maladaptive LV remodeling or dysfunction and higher event rates. These observations were the basis for our hypothesis that patients with higher biomarker levels, reflecting a sicker heart, would experience the greatest benefit from prompt unloading of the left ventricle. For the first time, we were able to test this hypothesis, given that patients were randomized to different strategies for AVR timing. Contrary to our hypothesis, there was no difference in the benefit observed from early TAVR across groups based on biomarker concentrations; in fact, the relative benefit of an early TAVR approach was numerically more pronounced (indicated by a lower HR) among patients with the lowest biomarker levels.

EVOLVED (Early Valve Replacement Guided by Biomarkers of LV Decompensation in Asymptomatic Patients With Severe AS), the results of which were reported recently,^16^ enrolled patients with asymptomatic severe AS with a detectable or elevated high-sensitivity troponin level (or LV hypertrophy on ECG) who were shown to have replacement fibrosis on cardiac magnetic resonance imaging. This was a reasonable study design to test optimal timing of AVR in an enriched, higher-risk cohort of patients based on circulating and imaging biomarkers. However, because of its design, that study was unable to test whether there was a differential response to early intervention based on baseline biomarker level (normal versus elevated), because only patients with replacement myocardial fibrosis, who tended to have elevated troponin levels and LV hypertrophy, were enrolled. By enrolling patients with normal and elevated NT-proBNP and hs-cTnT levels, EARLY TAVR provided an opportunity to evaluate whether there was a differential treatment effect across the spectrum of baseline biomarker levels.

### Clinical Implications and Future Directions

EARLY TAVR and other randomized trials testing optimal timing of AVR in patients with asymptomatic severe AS suggest that prompt intervention is superior to CS for these patients.^17,18^ The results of this biomarker substudy reinforce this broad approach and provide evidence (in the context of a randomized trial) that undercuts the guideline recommendation that AVR should be preferentially considered in the subset of asymptomatic patients with severe AS with an elevated BNP level.^1^ We tested this guideline recommendation explicitly and found that a natriuretic peptide level >3 times normal does not identify a subgroup of patients who experience greater benefit from early TAVR; instead, the relative benefit derived from early TAVR, compared with CS, was numerically greater in patients with a natriuretic peptide level ≤3 times normal. Contrary to our original hypothesis, we found that even if biomarker levels are low, clinical reassurance that waiting is safe is not necessarily warranted. Whereas event rates were lower in patients with lower biomarker levels and in many cases the absolute risk reduction from early TAVR in these patients was less and NNT larger, the relative benefit of prompt intervention was just as strong (even numerically stronger) among patients with lower biomarker levels compared with patients with higher biomarker levels and is the clinical pathway to the best outcome (ie, event rates were lowest among patients with low biomarker levels treated with early TAVR). If biomarker levels are elevated, there is even more urgency to intervene promptly given the higher event rates observed among these patients, the smaller NNT from prompt intervention in patients with higher NT-proBNP levels for end points including HFH, and the trend toward a greater likelihood of converting to AVR with a more acute and severe presentation.

For this biomarker substudy, it is worth considering how to understand and interpret the benefit of early TAVR, compared with CS, in terms of relative benefit (indicated by the HR) and absolute benefit (indicated by NNT). A relative benefit of early TAVR, compared with CS, was consistently observed for all biomarker subgroups for most outcomes, and the numerically lowest HR (indicative of greater relative benefit of early TAVR) was most commonly observed among patients with the lowest or normal biomarker levels. These findings ran counter to our original hypothesis. Even without a differential treatment effect based on baseline biomarker levels, biomarkers could point to patients with greater absolute risk reduction from early TAVR. In this regard, with respect to the absolute benefit of early TAVR, the findings were more mixed. In some cases (more commonly for NT-proBNP), because event rates were higher among patients with higher biomarker levels, the absolute benefit of early TAVR was greater and NNT lower among patients with higher biomarker levels despite the relative benefit being greater among patients with lower biomarker levels. For example, for the end point of HFH, the lowest tertile of NT-proBNP had a lower HR (0.00) for early TAVR (indicative of greater relative benefit) than the highest tertile of NT-proBNP (HR, 0.36), but there was a smaller absolute benefit of early TAVR among the lowest tertile of NT-proBNP (NNT=32) compared with the highest tertile of NT-proBNP (NNT=11). In some clinical scenarios, these differences in absolute risk reduction with an early TAVR approach based on a biomarker measurement could potentially inform decision making. However, in the context of this strategy trial on optimal timing of the TAVR procedure, NNT does not mean the number needed to treat (versus not treat) with the procedure to avoid one of the outcome events. Instead, it means the number of patients needed to treat with TAVR now versus treat with TAVR later (in EARLY TAVR, almost all CS patients were treated at a median of 11 months).^11^ Whether this tradeoff is meaningful may depend on the scenario.

After AS has progressed to the point at which a high gradient is present, prompt intervention yields a lower event rate for multiple end points across all biomarker subgroups. Whether this benefit of prompt intervention extends (whether broadly or more narrowly) to earlier stages of AS (ie, moderate AS), during which high event rates have also been observed, is unknown, and there is interest in identifying whether some patients may benefit from earlier AVR.^19,20^ In these patients with earlier-stage AS, it may be that only a subgroup will benefit from early intervention, rather than all. However, further study is warranted to answer this important clinical question. The heterogeneity of maladaptive LV remodeling or dysfunction at earlier stages of valve obstruction points to the potential utility of biomarkers to identify a subgroup of patients who may benefit from an early intervention strategy. This hypothesis will be tested in PROGRESS (PROGRESS: Management of Moderate Aortic Stenosis by Clinical Surveillance or TAVR), which also includes prospective collection of biospecimens.

### Limitations

Biospecimens were not collected in all patients in EARLY TAVR, but only 11% were missing, and these patients did not differ from patients with biospecimens collected (Table S8). Only patients ≥65 years of age (who are more likely to only need one procedure in their lifetime) were enrolled in EARLY TAVR and included in this biomarker substudy, but such patients represent the large majority of patients undergoing AVR. Just as with the full trial cohort, this subanalysis had a relatively lower percentage of women than expected on the basis of the sex distribution for TAVR procedures nationally, and, similar to national trends of patients undergoing TAVR, the percentage on non-White individuals was relatively low.^11^ EARLY TAVR was not specifically designed to test a biomarker-guided strategy for TAVR timing and the power to detect significant interactions between biomarker levels and treatment group allocation with respect to outcomes is limited. However, EARLY TAVR was a randomized controlled trial testing 2 distinct strategies with respect to timing of intervention, the biospecimens were collected prospectively, this analysis was prespecified, and the trends observed were in the opposite direction of our hypothesis. In accordance, there was no signal that higher biomarker levels might identify patients who would derive greater benefit from an early TAVR strategy compared with patients with lower biomarker levels. As opposed to a single cross-sectional measurement of biomarker concentrations, assessment of change over time from longitudinal measurements may have been informative, but the study design did not allow adequate time for serial collections before randomization.

### Conclusions

In this prespecified biomarker substudy of the EARLY TAVR randomized strategy trial, single baseline measurements of NT-proBNP and hs-cTnT levels did not modify the benefit of early TAVR for asymptomatic patients with severe high-gradient AS (stage C1). Whereas higher biomarker levels were associated with higher event rates, the relative benefit of an early TAVR strategy was consistent regardless of baseline biomarker concentrations, and biomarker concentrations did not meaningfully predict conversion timing to delayed AVR or provide reassurance that an acute or severe presentation could be avoided at conversion in the CS arm. An unexpected finding was that the relative beneficial effect of an early TAVR strategy tended to be greater (indicated by a lower HR) for both biomarkers and most end points examined among patients with the lowest, rather than highest, biomarker levels. Among all patients, those with the lowest biomarker concentrations assigned to the early TAVR arm had the best outcomes. In some cases, a higher NT-proBNP level identified patients who derived a greater absolute risk reduction with early TAVR, which may be useful for some clinical decisions on intervention timing. These findings reinforce the primary results of the EARLY TAVR trial and suggest limited value for single measurements of these biomarkers to guide the timing of TAVR in asymptomatic patients. Further studies are warranted to evaluate whether temporal changes in biomarker levels may inform optimal AVR timing and to elucidate whether cardiac biomarkers may identify a subgroup of patients with earlier-stage AS who may benefit from AVR before AS becomes severe.

## ARTICLE INFORMATION

### Acknowledgments

The authors thank Emma Robinson, PhD, an employee of Edwards Lifesciences, for editorial assistance.

### Sources of Funding

EARLY TAVR was funded by Edwards Lifesciences, Irvine, CA. The study was supported by grants R01HL164526 and R01AG073633 from the National Institutes of Health to Dr Lindman.

### Disclosures

Dr Lindman reports serving as a consultant for Anteris, AstraZeneca, Medtronic, and Kardigan; and serving as a consultant for and receiving institutional grant or contract support from Edwards Lifesciences. Dr Pibarot reports receiving institutional grant or contract support from Cardiac Success, Edwards Lifesciences, Medtronic, Novartis Pharma, and Pi-Cardiac. Dr Schwartz reports no disclosures. Dr Oldemeyer reports serving as a consultant for ABIOMED and as an investigator and on the case review and steering committees for Edwards Lifesciences. Dr Fearon reports receiving institutional grant or contract support from Abbott Vascular, CathWorks Inc, and Medtronic; holding stock options in HeartFlow Inc; being an employee of Veterans Affairs of Palo Alto; and serving as a consultant for ShockWave. Dr Babaliaros reports serving as a consultant for Abbott Vascular and Edwards Lifesciences and holding stock options in TransMural Systems. Dr Daniels reports serving as a consultant for Edwards Lifesciences. Dr Chhatriwalla reports serving as a consultant for Abbott Vascular, serving as a review committee member for ABIOMED, receiving grant or contract support from Boston Scientific Corporation, performing data and safety monitoring for Corematrix Cardiovascular, and serving as a consultant for and receiving travel honoraria from Edwards Lifesciences and Medtronic. Dr Suradi reports serving as a consultant for Edwards Lifesciences and Gore Medical. Dr Shah reports receiving institutional grant or contract support from Abbott Vascular and serving as a consultant for Boston Scientific Corporation, Edwards Lifesciences, and Xenter. Dr Szerlip reports being a steering committee member for Abbott Vascular, a speaker and advisory board member for Boston Scientific Corporation, a speaker for Edwards Lifesciences, and a steering committee member and advisory board member for Medtronic. Dr Mack reports being a trial coprincipal investigator for Edwards Lifesciences and a study chair for Medtronic. Dr Dahle reports being a speaker and proctor for Boston Scientific Corporation and Medtronic and a principal trial investigator and speaker and proctor for Edwards Lifesciences. Dr O’Neill reports serving as a consultant for Abbott Fund and Boston Scientific Corporation, receiving grant or contract support from ABIOMED, and serving as a consultant for and receiving grant or contract support from Edwards Lifesciences. Dr Davidson reports receiving institutional grant or contract support from Abbott Vascular, receiving institutional grant or contract support from and serving as an advisor for Edwards Lifesciences, and serving as a consultant for Philips. Dr Makkar reports receiving grant or contract support from Abbott Vascular and Edwards Lifesciences and serving as a consultant for Cordis Corporation and Medtronic. Dr Sheth reports receiving grant or contract support from Abbott Vascular and receiving institutional grant or contract support from and serving as a consultant for Edwards Lifesciences. Dr Depta reports serving as a consultant for Boston Scientific Corporation and Edwards Lifesciences. Dr DeVries has no relevant interests to disclose. Dr Southard reports serving as a proctor and consultant for Edwards Lifesciences. Dr Pop reports serving as a consultant for and receiving institutional grant or contract support from Edwards Lifesciences. Dr Sorajja reports serving as a consultant for 4C Medical Technologies, Abbott Structural, Adona Medical, Boston Scientific Corporation, CroiValve, Cultiv8, Edwards Lifesciences, Egg Medical, Evolution-Med, Foldax, GE Medical, Haemonetics, InQ8, Laza, Medtronic, Philips, Polares, W.L. Gore & Associates, vDyne, Unorthodox Ventures, Valcare Medical, and xDot. Dr Cohen reports serving as a consultant for and receiving institutional grant or contract support from Abbott Vascular, Boston Scientific Corporation, Edwards Lifesciences, and Medtronic, and receiving institutional grant or contract support from JenaValve. Dr Zhao is an employee of Edwards Lifesciences. Dr Goel reports serving as a consultant for Abbott Vascular and Edwards Lifesciences. Dr Su reports serving as the core laboratory director of and receiving institutional grant or contract support from Edwards Lifesciences. Dr Hahn reports serving as a speaker for Abbott Vascular, Baylis Medical Company, and Philipps; serving as the chief scientific officer of the echocardiographic core laboratory for the Cardiovascular Research Foundation; and serving as a speaker and echocardiographic data reviewer/adjudicator for Edwards Lifesciences. Dr Leon reports receiving institutional grant or contract support from Abbott Fund, ABIOMED, Anora Heart Inc, Boston Scientific Corporation, and Medtronic; serving as a consultant for Anteris, Croivalve, Foldax, Laminar, and MiRus LLC; serving as a principal investigator and receiving institutional grant or contract support from Edwards Lifesciences; holding stock options in Pi-Cardia and Xenter MD; and holding stock in SoloPace. Dr Généreux reports serving as a consultant for 4C Medical, Abbott Vascular, ABIOMED, Haemonetics Corporation, Medtronic, Opsens Inc, ShockWave Medical, and Teleflex Inc; serving as a principal investigator and consultant for Edwards Lifesciences; and serving as a consultant and holding stock options in Pi-Cardia, Puzzle Medical Inc, and Saranas Inc.

### Supplemental Material

Tables S1–S8

Figures S1–S9
